# COVID-19’s impact on hospital stays, mortality, and readmissions for poverty-related diseases, noncommunicable diseases, and injury groups in Thailand

**DOI:** 10.1371/journal.pone.0310090

**Published:** 2024-09-11

**Authors:** Satiti Palupi, Kyaw Ko Ko Htet, Vorthunju Nakhonsri, Chumpol Ngamphiw, Peerapat Khunkham, Sanya Vasoppakarn, Narumol Atthakul, Sissades Tongsima, Chantisa Keeratipusana, Watcharapot Janpoung, Virasakdi Chongsuvivatwong

**Affiliations:** 1 Department of Communicable Disease, East Java Provincial Health Office, Surabaya, Indonesia; 2 Department of Epidemiology, Faculty of Medicine, Prince of Songkla University, Hat Yai, Thailand; 3 National Biobank of Thailand, National Science and Technology Development Agency, Khlong Luang, Pathum Thani, Thailand; 4 Bureau of Service Quality Development, National Health Security Office, Lak Si, Bangkok, Thailand; Chiang Mai University, THAILAND

## Abstract

**Aims:**

This study aims to compare the trends in the quality of hospital care for WHO’s three disease groups pre-, during, and post-COVID-19 pandemic peak in Thailand.

**Methods:**

The study utilized existing hospital admission data from the Thai Health Information Portal (THIP) database, covering the period from 2017 to 2022. We categorized WHO’s three disease groups: poverty-related, noncommunicable, and injury groups using the International Classification of Diseases (ICD)—10 of initial admission of patients, and we analyzed three major outcomes: prolonged (≥ 90^th^ percentile) length of stay (LOS), hospital mortality, and readmission pre-, during, and post-COVID-19 pandemic peak. Relative weight (RW) of hospital reimbursements was used as a surrogate measure of the severity of the diseases.

**Results:**

The average prolonged LOS of patients with poverty disease pre-, during, and post-COVID-19 pandemic peak were 7.1%, 10.8%, 9.05%, respectively. Respective hospital mortality rates were 5.02%, 6.22%, 6.05% and readmission were 6.98/1,000, 6.16/1,000, 5.43/1,000, respectively. For non-communicable diseases, the respective proportions in the prolonged LOS were 9.0%, 9.12%, and 7.58%, with respective hospital mortality being 10.65%, 8.86%, 6.62%, and readmissions were 17.79/1,000, 13.94/1,000, 13.19/1,000, respectively. The respective prolonged LOS for injuries were 8.75%, 8.55%, 8.25%. Meanwhile, respective hospital mortality were 4.95%, 4.05%, 3.20%, and readmissions were 1.99/1,000, 1.60/1,000, 1.48/1,000, respectively. The RW analysis reveals diverse impacts on resource utilization and costs. Most poverty-related and noncommunicable diseases indicate increased resource requirements and associated costs, except for HIV/AIDS and diabetes mellitus, showing mixed trends. In injuries, road traffic accidents consistently decrease resource needs and costs, but suicide cases show mixed trends.

**Conclusions:**

COVID-19 had a more serious impact, especially prolonged LOS and hospital mortality for poverty-related diseases more than noncommunicable diseases and injuries.

## Introduction

On March 11, 2020, the World Health Organization (WHO) officially declared the COVID-19 pandemic as a worldwide crisis. This unprecedented event significantly affected various aspects of human life, particularly in the realm of healthcare. Essential health services were disrupted in 90% of countries across the world, with disruptions including resource constraints, reductions in elective procedures, and delays in essential care, as highlighted by the WHO [1–3]. These disruptions had profound implications for the quality of healthcare people received during the pandemic, as non-COVID-19 patients experienced a decreased quality of hospital care or outcomes [4].

The quality of hospital care is the degree of value attributed to a hospital, assessed through multiple measurements [5]. It is gauged by the effectiveness with which health services implement the most recent evidence-based professional knowledge and practices [6]. Three outcome measures commonly used in the USA, Netherlands, United Kingdom, Italy, Belgium, and Australia to evaluate the quality of care in hospitals are prolonged length of stay (LOS), hospital mortality, and readmission [7].

The COVID-19 pandemic also has significantly impacted Thailand’s health service, as it has in many other countries worldwide. A prior study revealed that health facilities in Bangkok had to cope with a particularly high number of COVID-19 cases during the pandemic, which might affect the quality of hospital care [8]. This highlights the importance of health service resilience to ensure the continuation of health facility services after the pandemic.

As of October 20, 2023, the Thai government has reported a total of 4,757,728 confirmed cases of COVID-19 [9]. Thailand’s first wave of COVID-19 in March 2020, peaked on March 22, 2020. Subsequently, on March 26, 2020, the Thai government declared a state of emergency in response [10]. On October 1, 2022, the Thai government announced the end state of COVID-19 as an emergency disease or dangerous communicable disease into a communicable disease under surveillance [11].

To comprehensively understand the pandemic’s impact on healthcare quality, this study aims to compare trends in hospital care quality for WHO’s three disease groups pre-, during, and post-COVID-19 pandemic peak in Thailand.

## Materials and methods

### Study design and data source

The study design involved a series of analyses of existing data in the Thai Health Information Portal (THIP) database from 2017 to 2022.

THIP is a cooperative data warehouse project managed jointly by the National Health Security Office (NHSO) of Thailand, Prince of Songkla University, and the National Science and Technology Development Agency (NSTDA) of Thailand. The project retrieved claimed data from patient files at NHSO and NSTDA stores and saved the data to the user. PSU acts as the gatekeeper [12]. The information is used to support education and research. All records are de-identified.

In this study, THIP data was accessed between September 5, 2023, and December 16, 2023. The Institutional Ethics Committee of the Faculty of Medicine at Prince of Songkla University in Hat Yai, Thailand, granted ethical approval under reference number REC 66-485-18-1. Prof. Boonsin Tangtrakulwanich, Chairman of the Human Research Ethics Committee, Faculty of Medicine, Prince of Songkla University, approved the study.

The variables we used were encrypted personal ID, sex, age, admission date, discharge date, primary diagnosis (based on the International Classification of Diseases (ICD-10)), discharge status, and Relative Weight (RW) of DRG (diagnosis-related groups).

The diagnosis-related groups (DRG) prospective payment system was introduced under the Universal Health Coverage (UHC) scheme in Thailand to standardize reimbursement payments for inpatient hospital admissions, including those for cancer patients. This system categorizes diseases with similar characteristics into diagnostic and treatment groups to ensure uniformity in clinical processes and resource consumption costs. The relative weights (RW) of each DRG were determined based on existing hospital charges and health insurance reimbursement [13]. An elevated RW indicates increased resource requirements for treating a patient, culminating in higher medical costs [14]. Since the hospital resources used for COVID-19 diseases might vary over the study period, in comparison to the outcome, we used RW to make sure the effects of the period are comparable.

### Study population and time period

The study population was all patients admitted to Thailand hospitals between 2017 and 2022.

### Diseases groups

In this study, to assess the quality of hospital care for WHO’s three disease groups, we categorized various diseases into three major groups: poverty-related diseases, noncommunicable diseases, and injuries [15, 16].

In this study, we used diarrhea, HIV/AIDS, influenza, pneumonia, and tuberculosis (TB), which was a highly spread infectious disease and was also a poverty-related disease as a representative of the poverty-related diseases group [15, 17, 18]. The noncommunicable disease group, we used cancer, chronic obstructive pulmonary disease (COPD), ischemic heart disease, hypertensive disease, stroke, and diabetes mellitus as representatives of the noncommunicable diseases group [15, 19], which was common noncommunicable diseases in the world and also associated with increased risk of complications and mortality from COVID-19 [19] and we explored road traffic accidents and suicide as representatives of unintentional injuries and intentional injuries in the injuries group [15, 20]. Period pre-, during and post-COVID-19 pandemic.

**Fig 1:**
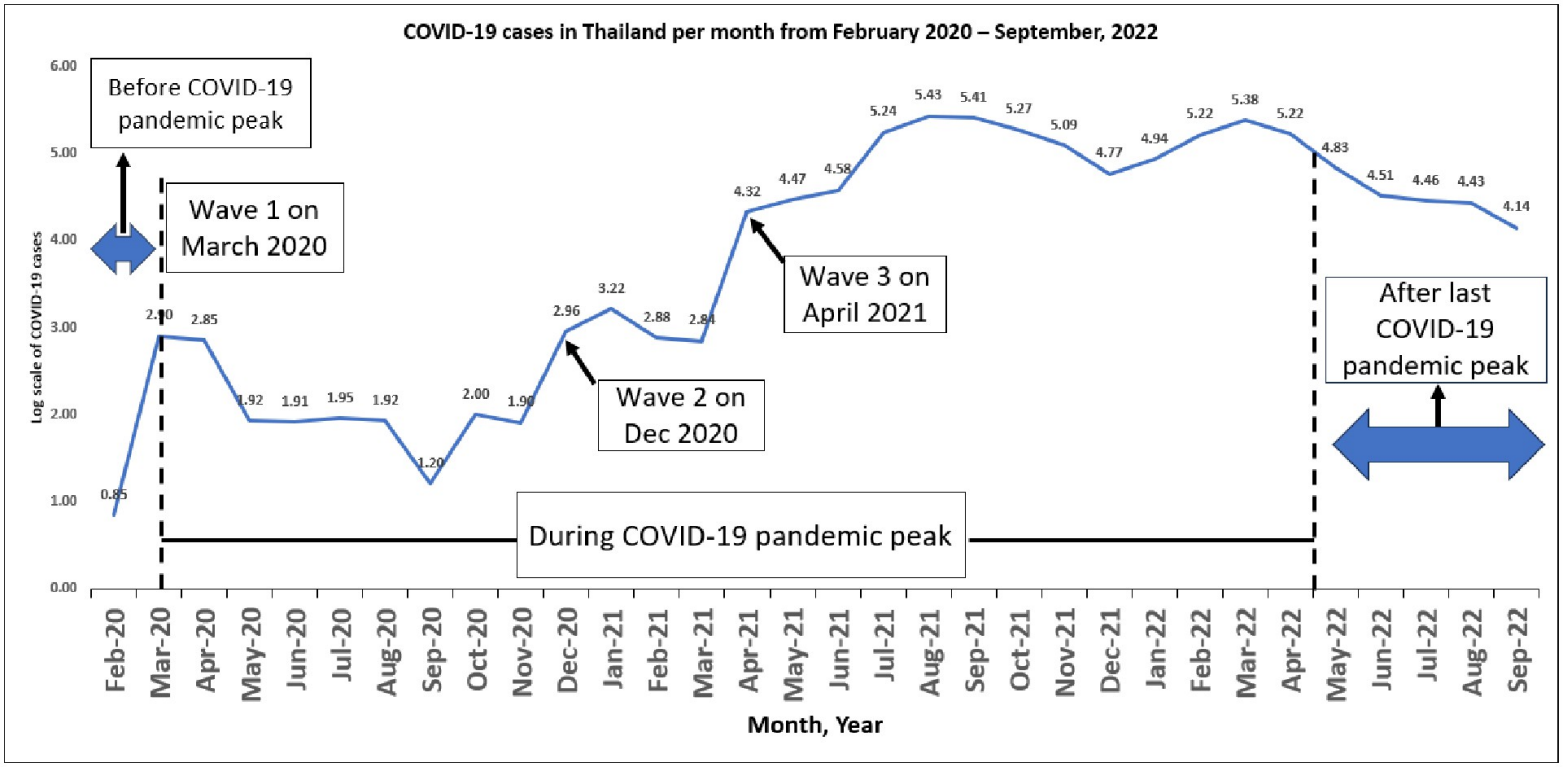
Fluctuations in COVID-19 cases in Thailand from time to time (February 22, 2020–September 30, 2022).

Fig 1 illustrates the trend of COVID-19 cases on a logarithmic scale. The study timeline was categorized into three phases: the pre-COVID-19 pandemic peak period (prior to March 2020) encompassing a time without implemented public health measures, the during-COVID-19 pandemic peak period (up to April 2022), marked by diverse public responses such as national lockdowns and hospital use restrictions; and the post-COVID-19 pandemic peak period, denoting a phase after the lifting of all implemented measures.

### ICD-10 grouping

Table 1 lists disease codes grouped into three categories.

**Table 1:** ICD-10 codes of the diseases.

Group	Diseases	Subgroup	ICD-10 codes
**Poverty-related diseases**	Diarrhea		A00, A01, A03, A04, A06-A09
	HIV/AIDS		B20–B24
	Influenza	Lower respiratory infection	J09–J11
	Pneumonia	Lower respiratory infection	J12-J18
	Tuberculosis (TB)		A15–A19
**Noncommunicable disease**	Cancer		C00–C97
	Chronic Obstructive Pulmonary Disease (COPD)		J40–J47
	Ischemic heart disease	Chronic Vascular Disease (CVD)	I20–I25
	Hypertensive disease	Chronic Vascular Disease (CVD)	I10–I13
	Stroke	Chronic Vascular Disease (CVD)	I63, I65, I66
	Diabetes mellitus (DM)		E10–E14
**Injuries**	Road traffic accident		V01–V99
	Suicide		X60–X84

### Outcomes

Length of stay (LOS) was the length of time elapsed between a patient’s hospital admittance and discharge. The average length of stay (LOS) was calculate for individual hospitalization, greater and equal to the 90^th^ percentile for the specific diagnosis or procedure group (upper bound of outlier length of stay) is considered as prolonged LOS [7, 21]. Hospital mortality is defined as death from any cause in the hospitalized population, recorded in by hospital discharge status [22, 23]. Finally, readmission is defined as unplanned subsequence acute care inpatient admission of the same patient within 72 hours of discharge of the initial inpatient acute care admission [24, 25].

### Statistical analysis

The data will be analyzed by using R version 3.1.1 (R Computing, Vienna, Austria).

Main outcome variables include whether the patients had the specific outcome at discharge time. Main independent variable included the period (pre-COVID-19 pandemic peak, during COVID-19 pandemic peak and post-COVID-19 pandemic peak). Covariates used for adjustment include age, sex, and RW.

Univariate analyses were used to scan the association between the independent and the outcome variables. Then we conducted logistic regression to assess the differences in COVID-19 pandemic peak period (pre-, during, and post-) in relation to prolonged length of stay, hospital mortality, and readmission in each disease with adjustment of sex, age, and RW.

## Results

### General description

From 2017 to 2022, altogether, 4,819,921 patients were admitted from 2017, to 2022, with ICD-10 codes of interest in primary diagnoses. Table 2 shows data on sex and age distribution across different time periods (pre-, during, and post-COVID-19 pandemic peak). In this study, 54.75% patients were males. The mean age was higher during COVID-19 pandemic peak than other periods.

**Table 2:** Baseline characteristics of admitted patients in hospital.

Period of COVID-19 pandemic peak				
Variables	Pre-	During	Post-	Overall
**Sex**	(N = 2,376,649)	(N = 1,418,160)	(N = 273,976)	(N = 4,068,790)
Female	1,021,471	603,555	118,297	1,743,323
Male	1,355,178	814,605	155,679	2,325,462
**Age (year)**				
Mean (SD)	58.8 (± 20.5)	59.1 (±19.6)	59.0 (±19.7)	58.9 (± 20.2)
Median (min, max)	63.0 (0, 117)	63.0 (0, 122)	63.0 (0, 117)	63.0 (0, 122)
**Poverty-related diseases group**				
Diarrhea	233,011	108,582	20,845	362,438
HIV/AIDS	10,878	5,502	1,040	17,420
Lower respiratory Infection (LRI)	972,797	745,374	134,790	1,852,961
Tuberculosis (TB)	101,763	59,920	10,613	172,296
**Non-communicable diseases group**				
Cancer	302,293	196,676	38,486	537,455
Chronic Obstructive Pulmonary Disease (COPD)	162,678	58,361	12,454	233,493
Cardiovascular disease (CVD)	631,170	388,910	75,595	1,095,675
Diabetes mellitus (DM)	42,932	32,798	6,440	82,170
**Injuries group**				
Road traffic accident	224,446	152,025	26,478	402,949
Suicide	36,308	26,479	6,432	69,219

### Prolonged length of stay

Table 3 shows the 90^th^ percentile of length of stay of various diseases in descending order, note that TB ranked the second only CVD.

**Table 3:** 90^th^ percentile of length of stay of various diseases of interest.

Diseases	90^th^ percentile of length of stay (days)
**Poverty-related diseases group**	
Diarrhea	5
HIV/AIDS	13
LRI	14
TB	17
**Non-communicable diseases group**	
Cancer	14
COPD	9
CVD	37
DM	5
**Injuries group**	
Road traffic accident	5
Suicide	5

### Poverty-related diseases group

Table 4 provides a comprehensive overview indicating a consistent rise in both the percentage and average percentage of prolonged LOS during and post-COVID-19 pandemic peak within the poverty-related diseases group. Concurrently, there was a consistent upward trend in the percentage of hospital mortality in this group, in line with an increase in the average percentage, except for HIV/AIDS, which displayed mixed trends. Conversely, most readmissions in poverty-related diseases decreased during and post-COVID-19 pandemic peak, aligning with the overall decrease in the average per thousand patient readmissions in this group. However, HIV/AIDS presented mixed trends in this context.

**Table 4:** Percentages of prolonged length of stay and hospital mortality, per thousand readmission, and relative weight of poverty-related diseases group between pre-, during and post-COVID-19 pandemic peak.

Outcomes of poverty-related diseases group	Period of COVID-19 pandemic peak			P value
	Pre-	During	Post-	
**Prolonged length of stay**	**Percentage (%)**	**Percentage (%)**	**Percentage (%)**	
Diarrhea	7.8	9.8	10.2	<0.001
HIV/AIDS	9.8	11.2	10.3	0.0155
LRI	0.5	10.7	5.2	<0.001
TB	10.3	11.6	10.5	<0.001
**Average**	**7.1**	**10.8**	**9.05**	**<0.001**
**Hospital mortality**	**Percentage (%)**	**Percentage (%)**	**Percentage (%)**	
Diarrhea	1.2	1.5	1.4	<0.001
HIV/AIDS	7.8	8.6	7.5	<0.001
LRI	3.5	5.6	6.2	0.4009
TB	7.6	9.2	9.1	<0.001
**Average**	**5.025**	**6.22**	**6.05**	**<0.001**
**Readmission**	**Per thousand**	**Per thousand**	**Per thousand**	
Diarrhea	2.34	2.07	1.54	0.0072
HIV/AIDS	8.22	10.29	7.34	0.4319
LRI	4.70	2.09	2.85	<0.001
TB	12.65	10.18	9.99	<0.001
**Average**	**6.98**	**6.16**	**5.43**	**<0.001**
**Relative weight (RW)**	$\bar{\text{x}}\pm \text{SD}$	$\bar{\text{x}}\pm \text{SD}$	$\bar{\text{x}}\pm \text{SD}$	
Diarrhea	0.426 ± 0.65	0.473 ± 0.744	0.471 ± 0.652	<0.001
HIV/AIDS	1.244 ± 1.319	1.285 ± 1.448	1.227 ± 1.072	0.0753
LRI	1.016 ± 2.21	1.161 ± 2.139	1.359 ± 2.215	<0.001
TB	1.624 ± 2.293	1.848 ± 2.485	1.853 ± 2.22	<0.001

The relative weights (RW) for diarrhea, LRI, and TB show consistently increased during, and post- compared with pre-COVID-19 pandemic peak. These changes indicate an increase in resource requirements and average costs associated with severe illness of patients with these diseases.

The results of logistic regression in S1 Table, predicting prolonged length of stay, hospital mortality, and readmission of poverty-related diseases with adjustment for sex, age, and RW, were similar to the result in Table 4 (without the adjustment).

### Noncommunicable diseases group

Table 5 summarizes that the percentage of prolonged LOS during and post-COVID-19 pandemic peak consistently increased in COPD, decreased in cancer, and had mixed trends in CVD and DM compared to pre-COVID-19 pandemic peak. The average percentages of prolonged LOS also exhibited mixed trends. Concurrently, hospital mortality consistently decreased, along with readmissions during and post-COVID-19 pandemic peak, aligning with the observed patterns in the average percentages of hospital mortality and readmissions per thousand in this group.

**Table 5:** Percentages of prolonged length of stay and hospital mortality, per thousand readmission, and relative weight of noncommunicable diseases group between pre-, during, and post-COVID-19 pandemic peak.

Outcomes of noncommunicable diseases group	Period of COVID-19 pandemic peak			P value
	Pre-	During	Post-	
**Prolonged length of stay**	**Percentage (%)**	**Percentage (%)**	**Percentage (%)**	
Cancer	9.7	8.3	7.4	<0.001
COPD	7.7	9.1	8.4	<0.001
CVD	10.0	10.9	5.9	0.0079
DM	8.6	8.2	8.6	0.0181
**Average**	**9.0**	**9.12**	**7.58**	**<0.001**
**Hospital mortality**	**Percentage (%)**	**Percentage (%)**	**Percentage (%)**	
Cancer	19.8	16.2	11.2	<0.001
COPD	8.3	6.8	5.5	<0.001
CVD	8.5	8.3	7.3	<0.001
DM	6.0	4.2	2.5	<0.001
**Average**	**10.65**	**8.87**	**6.62**	**<0.001**
**Readmission**	**Per thousand**	**Per thousand**	**Per thousand**	
Cancer	23.77	18.15	15.92	<0.001
COPD	33.54	24.95	25.16	<0.001
CVD	8.75	8.15	7.16	<0.001
DM	5.09	4.49	4.53	0.0408
**Average**	**17.79**	**13.94**	**13.19**	**<0.001**
**Relative weight (RW)**	** $\bar{\text{x}}\pm \text{SD}$ **	$\bar{\text{x}}\pm \text{SD}$	$\bar{\text{x}}\pm \text{SD}$	
Cancer	2.742 ± 2.336	2.805 ± 2.262	2.803 ± 2.17	<0.001
COPD	1.396 ± 2.392	1.478 ± 2.4	1.536 ± 2.188	<0.001
CVD	3.532 ± 4.265	3.835 ± 4.409	3.833 ± 4.284	<0.001
DM	2.262 ± 3.248	2.345 ± 3.173	2.208 ± 2.653	<0.001

The relative weights for cancer, COPD, and CVD consistently increased during, and post- compared with pre-COVID-19 pandemic peak. These changes indicate an increase in resource requirements and average costs associated with severe illness of patients with these diseases. Meanwhile, DM shows mixed trends.

As shown in S2 Table, logistic regression with adjustment for sex, age, and RW had similar results to those in Table 5 (without the adjustment).

### Injuries group

Table 6 provides a summary of the percentage of prolonged length of stay (LOS) during and post-COVID-19 pandemic peak, revealing mixed trends within the injuries group when compared to the pre-COVID-19 pandemic peak. On the other hand, the average percentage of prolonged LOS demonstrated a decrease during and post-COVID-19 pandemic peak. Meanwhile, hospital mortality consistently decreased in cases of suicide during, and post-COVID-19 pandemic peak compared to the pre-COVID-19 pandemic peak period, along with a reduction in readmissions. This aligns with the observed patterns in the average percentage of hospital mortality and per thousand readmission in this context.

**Table 6:** Percentages of prolonged length of stay and hospital mortality, per thousand readmission, and relative weight of injuries group between pre-, during, and post-COVID-19 pandemic peak.

Outcomes of injuries group	Period of COVID-19 pandemic peak			P value
	Pre-	During	Post-	
**Prolonged length of stay**	**Percentage (%)**	**Percentage (%)**	**Percentage (%)**	
Road traffic accident	8.6	8.2	8.6	0.0181
Suicide	8.9	8.9	7.9	0.0422
**Average**	**8.75**	**8.55**	**8.25**	
**Hospital mortality**	**Percentage (%)**	**Percentage (%)**	**Percentage (%)**	
Road traffic accident	3.9	3.9	3.9	0.2981
Suicide	6.0	4.2	2.5	<0.001
**Average**	**4.95**	**4.05**	**3.2**	
**Readmission**	**Per thousand**	**Per thousand**	**Per thousand**	
Road traffic accident	2.13	1.96	2.37	0.856
Suicide	1.85	1.25	0.59	0.0045
**Average**	**1.99**	**1.61**	**1.48**	
**Relative weight (RW)**	$\bar{\text{x}}\pm \text{SD}$	$\bar{\text{x}}\pm \text{SD}$	$\bar{\text{x}}\pm \text{SD}$	
Road traffic accident	2.932 ± 4.09	2.841 ± 3.844	2.727 ± 3.451	<0.001
Suicide	0.512 ± 1.271	0.528 ± 1.317	0.476 ± 0.983	0.0079

The relative weights for road traffic accidents show consistently decreased during and post- compared with pre-COVID-19 pandemic peak. These changes indicate a reduction in resource requirements and average costs associated with severe illness of patients with these diseases. Meanwhile, suicide shows mixed trends.

The S3 Table shows logistic regression results after adjustment for sex, age, and RW, which are similar to those in Table 6 (without the adjustment).

We summarize the direction of effect and consistent findings among three major disease groups with the quality of hospital care outcomes as shown in Table 7. Poverty-related diseases exhibit challenges, with consistently increased prolonged length of stay and hospital mortality. Noncommunicable diseases present mixed trends in prolonged length of stay, but consistently show improvements with decreased hospital mortality and readmissions.

**Table 7:** Direction of effect and consistent findings among disease groups and the outcomes of quality of hospital care.

Groups	Poverty-related diseases	Noncommunicable diseases	Injuries
**The severity of illness as reflected by relative weight (RW) of hospital reimbursement**	Mostly increase consistently	Mostly increase consistently	Road traffic injuries RW decrease consistently. Suicide RW increased during COVID pandemic peak
**Outcomes**			
Prolonged length of stay	Increase consistently	Mixed trend	Mixed trend
Readmission	Mostly decrease consistently	Decrease consistently	Readmission of road traffic injuries did not significantly.
			Suicide decreases in readmission
Hospital mortality	Mostly increase consistently	Decrease consistently	Hospital mortality of road traffic injuries did not change significantly.
			Suicide decreases in hospital mortality

## Discussion

The severity of poverty-related diseases and noncommunicable diseases increased consistently. The prolonged hospital stay was observed mainly in cases of poverty-related diseases. Hospital mortality increased consistently in the diseases of poverty-related diseases but decreased consistently in noncommunicable diseases and suicide. Finally, readmission decreases consistently in nearly all disease groups.

The RW analysis highlights varying impacts on resource utilization and costs associated with severe illness across different diseases. An upward trend for most diseases in poverty-related diseases and noncommunicable diseases signaling increased resource requirements and associated costs linked to severe illness.

It might be due to the fact that the COVID-19 pandemic deepened the deprivation of low socioeconomic status (SES) groups [26, 27], which then led to more complications, such as the diseases of poverty. Furthermore, the COVID-19 pandemic might delay decisions and facilities of low SES groups in arriving at the health service timely. These phenomena were likely in chronic noncommunicable diseases, the diseases in which groups progress more slowly, and in road traffic accidents where prompt response by the rescue system was not much disrupted by the pandemic. The same explanation could be applied to prolonged length of stay.

Effects on hospital readmission were quite different from that on length of stay. In general, readmission is partially affected too early discharge [28], when the final diagnosis and/or treatment were not reached. The COVID-19 pandemic decreased the hospital’s general caseload due to the lockdown process and people who were generally scared of contracting the infection. Reduction of the general caseload could reduce the need for early discharge of the patients to reduce hospital congestion reduction of early discharge, thus reducing the chance of readmission.

Across various disease groups, poverty-related diseases generally showed an increase in both prolonged LOS and hospital mortality during and post-pandemic periods. HIV/AIDS was the exception, which exhibited mixed trends. Conversely, readmissions for poverty-related diseases generally decreased during and post-COVID-19 pandemic peak. Similar trends were observed in noncommunicable diseases, where COPD demonstrated an increased prolonged LOS, cancer consistently decreased, and hospital mortality decreased across various diseases. In the injuries group, the study found mixed trends in the percentage of prolonged LOS, but hospital mortality consistently decreased, particularly in cases of suicide, and readmissions declined.

Respiratory distress and gastrointestinal symptoms are common in many infectious diseases such as COVID-19, influenza, pneumonia, and even TB [29, 30]. To rule out COVID-19 transmission in the pandemic period, the hospitalization period needed to be extended, which led to the lengthening of LOS. Patients might have prolonged delays in arriving at the hospital to get service due to worrying about getting a COVID-19 infection from the hospital and inconvenience in transportation and the referral system. These delays would increase the clinical complication level, leading to extended LOS. The change of pattern in healthcare systems during the pandemic could result in delayed access to medical care, leading to more severe presentations of these conditions when patients finally seek treatment [31].

Lockdowns may limit people’s ability to seek timely healthcare for various conditions, leading to more severe presentations when they eventually seek treatment. In addition, during the pandemic, delayed diagnosis of new cases often led to severe disease complications [32]. These new cases of complications are also probably accumulated in the post-pandemic period, particularly for poverty-related diseases such as TB, respiratory diseases, and HIV. Although delayed diagnosis of new cases can also occur in COPD and cancer patients, the slow progression of these new diseases means they are less likely to present as severe cases in the post-pandemic period. More severe cases often require longer hospital stays or hospital mortality. More severe cases often require longer hospital stays or hospital mortality [33–35].

Besides that, COVID-19 could cause severe respiratory complications, and individuals with pre-existing conditions, such as HIV, influenza, pneumonia, and TB, were more susceptible to these complications. However, the magnitude of these problems is not known.

People were worried about contracting COVID-19 while in a healthcare setting. Hospitals were perceived as places where the risk of exposure to the virus may be elevated [36]. According to several studies, many people in the USA and other countries have delayed or avoided medical care during the pandemic due to fear of COVID-19, financial difficulties, or lack of access [37–39]. This could lead to worse health outcomes for those with chronic or acute conditions [40, 41].

Prolonged LOS consistently increased during and post-COVID-19 pandemic peak for COPD, potentially due to the similarities in clinical presentation between COPD exacerbations and COVID-19 [42, 43]. This overlap in symptoms may have led to challenges in differentiating between the two conditions, potentially delaying appropriate treatment and contributing to longer hospital stays. Additionally, the pandemic may have strained healthcare resources, limiting the availability of specialized care for COPD patients and further contributing to prolonged LOS [44].

Based on previous studies, there are home chemotherapy programs in Thailand, which may be connected to adaptations in healthcare delivery during lockdowns [45]. The mention of a home chemotherapy program in Thailand suggests a shift towards more efficient and possibly remote care delivery, which could be a response to the limitations imposed by lockdown policies. Previous research also conducted in Thailand during the pandemic showed that DM patients could be resilient using cost-effective and standard digital technologies to provide a continuum of care for DM patients, and alternative services like mobile medical labs, medication delivery, and medical refills at drug stores can improve continuous monitoring of glycemic control and prescribed medication use [8]. Thailand also has had a universal health care program that eases the impact of DM since pre-COVID-19 pandemic. This has caused the Thai government to be well-prepared to handle DM during and post-COVID-19 pandemic [46, 47]. Reduced mortality was observed in patients with noncommunicable diseases during and post-COVID-19 pandemic peak. This may reflect success in the Thai health system. In Thailand, health programs adopted care models to ensure the safety of care for people living with noncommunicable diseases during the pandemic [48].

The observed decrease in readmissions within three days for both poverty-related diseases and noncommunicable diseases, as well as for cases of suicide. These findings may be indicative of changes in healthcare utilization patterns, possibly influenced by pandemic-related factors such as altered healthcare-seeking behavior, increased awareness of preventive measures, or changes in healthcare delivery systems [49–51].

Poverty-related diseases have higher mortality rates during the pandemic due to increased exposure, vulnerability and barrier to health care of low socioeconomic status groups during the pandemic [52]. On the other hand, the decrease in hospital mortality rates in NCDs may be due to the increased focus on preventive measures and early detection of NCDs during the pandemic [53, 54].

The decrease in injuries hospital mortality in suicide may be due to the increased awareness of mental health issues during the pandemic. This study result was in line with the previous study in the USA that suicide deaths between 2019 and 2020 decreased by 3% overall [55]. In the lockdown period, although depression could become more common, people are confined together in the same premises. This may lessen the chance of an individual to make a commit suicide. Supportive relationships and mental health interventions played crucial roles in suicide prevention [56].

These findings provide a nuanced understanding of the pandemic’s multifaceted impact on hospital care quality across different disease categories in the Thai healthcare system, offering valuable insights for future healthcare planning and improvements.

Furthermore, using relative weight (RW) as a surrogate measure of disease severity provides a novel perspective on the pandemic’s economic impact on healthcare resource utilization and costs, adding a unique dimension to the existing literature.

This study has several limitations. First, it relies on hospital admission data, which may not fully capture the broader health outcomes of the entire population. Second, the data lacks information on disease severity, which could be a potential confounder in the analysis. Third, the use of relative weight (RW) as a surrogate measure of disease severity has its limitations, as it may not fully reflect the complexity and heterogeneity of individual cases. Finally, the study is observational. That limitation emphasizes the need for future research to adopt a more holistic approach to understanding the comprehensive implications of healthcare changes during and post-COVID-19, incorporating community-level health indicators and qualitative assessments alongside quantitative data. This research should investigate the long-term impact of the pandemic on hospital care quality, examine the effects on specific subpopulations, and explore health disparities, the role of healthcare infrastructure, and the effectiveness of public health interventions. Comparative studies across different countries could provide valuable insights for global pandemic preparedness and response.

The results of this study have several implications for healthcare policy and resource allocation in Thailand: prioritizing cases for vulnerable populations, enhancing healthcare infrastructure, optimizing resource allocation, and strengthening pandemic preparedness.

## Conclusions

COVID-19 had a more serious impact, especially prolonged LOS, and hospital mortality for poverty-related diseases more than noncommunicable diseases and injuries. More attention should therefore be given to improving care to the poor.

## Supporting information

S1 Table Logistic regression analysis of the association between prolonged length of stay, hospital mortality, and readmission of poverty-related diseases group and period of COVID-19 pandemic peak with adjustment for sex, age, and RW. (DOCX)

S2 Table Logistic regression analysis of the association between prolonged length of stay, hospital mortality, and readmission of noncommunicable diseases group and period of COVID-19 pandemic peak with adjustment for sex, age, and RW. (DOCX)

S3 Table Logistic regression analysis of the association between prolonged length of stay, hospital mortality, and readmission of injuries group and period of COVID-19 pandemic peak with adjustment for sex, age, and RW. (DOCX)

## References

[1] Huggins A, Husaini M, Wang F, Waken R, Epstein AM, Orav EJ, et al. Care Disruption During COVID-19: a National Survey of Hospital Leaders. J Gen Intern Med. 2023; 38(5):1232–8 doi: 10.1007/s11606-022-08002-5 . 36650332 PMC9845025

[2] World Health Organization. COVID-19 continues to disrupt essential health services in 90% of countries. 2021. https://www.who.int/news/item/23-04-2021-covid-19-continues-to-disrupt-essential-health-services-in-90-of-countries.

[3] World Health Organization. Essential health services face continued disruption during COVID-19 pandemic. 2022. https://www.who.int/news/item/07-02-2022-essential-health-services-face-continued-disruption-during-covid-19-pandemic.

[4] Sabetkish N, Rahmani A. The overall impact of COVID-19 on healthcare during the pandemic: A multidisciplinary point of view. Health Sci Rep. 2021; 4(4):e386. doi: 10.1002/hsr2.386 34622020 PMC8485600

[5] Agency for Healthcare Research and Quality. Measuring the Quality of Hospital Care. 2016. https://www.ahrq.gov/talkingquality/measures/setting/hospitals/index.html.

[6] World Health Organization. Quality of care. 2024. https://www.who.int/health-topics/quality-of-care.

[7] Lingsma HF, Bottle A, Middleton S, Kievit J, Steyerberg EW, Marang-van de Mheen PJ. Evaluation of hospital outcomes: the relation between length-of-stay, readmission, and mortality in a large international administrative database. BMC Health Serv Res. 2018; 18:116. doi: 10.1186/s12913-018-2916-1 . 29444713 PMC5813333

[8] Pitayarangsarit S, Bhagaman N, Yodmai K, Thangsirikul N, Tipayamongkholgul M. The resiliency of noncommunicable diseases services during the public health crisis: a lesson from Bangkok, Thailand. BMC Health Serv Res. 2023; 23(1):409. doi: 10.1186/s12913-023-09400-z . 37101168 PMC10132400

[9] Worldometer. COVID—Coronavirus Statistics. 2023. https://www.worldometers.info/coronavirus/

[10] Kunno J, Supawattanabodee B, Sumanasrethakul C, Wiriyasivaj B, Kuratong S, Kaewchandee C. Comparison of Different Waves during the COVID-19 Pandemic: Retrospective Descriptive Study in Thailand. Adv Prev Med. 2021; 2021:5807056. doi: 10.1155/2021/5807056 . 34659835 PMC8519693

[11] Bangkok Post. End of state of emergency and Covid-19 centre. 2022. https://www.bangkokpost.com/thailand/general/2398843/end-of-state-of-emergency-and-covid-19-centre

[12] Sirichamratsakul K, Laochareonsuk W, Surachat K, Sangkhathat S. Population-based prevalence study of common congenital malformations of the alimentary tract and abdominal wall in Thailand: a study using data from the National Health Security Office. World J Pediatr Surg. 2023; 6(3):e000540. doi: 10.1136/wjps-2022-000540 . 37303481 PMC10254801

[13] Bredenkamp C, Bales S, Kahur K. Transition to Diagnosis-Related Group (DRG) Payments for Health: Lessons from Case Studies. World Bank. 2020. https://openknowledge.worldbank.org/handle/10986/33034.

[14] Tangcharoensathien V, Limwattananon S, Patcharanarumol W, Thammatacharee J, Jongudomsuk P, Sirilak S. Achieving universal health coverage goals in Thailand: the vital role of strategic purchasing. Health Policy Plan. 2015; 30(9):1152–61. doi: 10.1093/heapol/czu120 . 25378527 PMC4597041

[15] World Health Organization. List of causes and corresponding ICD-10 codes. 2023. https://platform.who.int/mortality/about/list-of-causes-and-corresponding-icd-10-codes.

[16] Emadi M, Delavari S, Bayati M. Global socioeconomic inequality in the burden of communicable and non-communicable diseases and injuries: an analysis on global burden of disease study 2019. BMC Public Health. 2021; 21(1):1771. doi: 10.1186/s12889-021-11793-7 . 34583668 PMC8480106

[17] Mondial Diagnostics. Poverty-Related Diseases. 2021. https://www.mondialdx.com/mondialdx.com/index.php/poverty-related-diseases.

[18] Dorlo TPC, Fernández C, Troye-Blomberg M, de Vries PJ, Boraschi D, Mbacham WF. Poverty-Related Diseases College: a virtual African-European network to build research capacity. BMJ Global Health. 2016;1(1):e000032. doi: 10.1136/bmjgh-2016-000032 . 28588923 PMC5321328

[19] World Health Organization. Non communicable diseases. 2023. https://www.who.int/news-room/fact-sheets/detail/noncommunicable-diseases.

[20] World Health Organization. Injuries and violence. 2021. https://www.who.int/news-room/fact-sheets/detail/injuries-and-violence.

[21] Analytics Vidhya. Outlier Detection & Removal | How to Detect & Remove Outliers. 2021. https://www.analyticsvidhya.com/blog/2021/05/feature-engineering-how-to-detect-and-remove-outliers-with-python-code/.

[22] Asch DA, Sheils NE, Islam MN, Chen Y, Werner RM, Buresh J, et al. Variation in US Hospital Mortality Rates for Patients Admitted With COVID-19 During the First 6 Months of the Pandemic. JAMA Intern Med. 2021; 181(4):471. doi: 10.1001/jamainternmed.2020.8193 . 33351068 PMC7756246

[23] The Centers for Disease Control and Prevention. COVID-19 and Cancer Deaths. 2023. https://www.cdc.gov/cancer/dcpc/research/articles/covid-19-cancer-deaths.html.

[24] Graham KL, Wilker EH, Howell MD, Davis RB, Marcantonio ER. Differences Between Early and Late Readmissions Among Medical Patients, A Cohort Study. Ann Intern Med. 2015; 162(11):741–9. . 26030632 10.7326/AITC201506020 PMC4747330

[25] Ramos-Martínez A, Parra-Ramírez LM, Morrás I, Carnevali M, Jiménez-Ibañez L, Rubio-Rivas M, et al. Frequency, risk factors, and outcomes of hospital readmissions of COVID-19 patients. Sci Rep. 2021; 11(1):13733. doi: 10.1038/s41598-021-93076-0 . 34215803 PMC8253752

[26] Anderson G, Frank JW, Naylor CD, Wodchis W, Feng P. Using socioeconomics to counter health disparities arising from the COVID-19 pandemic. BMJ. 2020; m2149. doi: 10.1136/bmj.m2149 . 32513666

[27] Naylor-Wardle J, Rowland B, Kunadian V. Socioeconomic status and cardiovascular health in the COVID-19 pandemic. Heart. 2021; 107(5):358–65. doi: 10.1136/heartjnl-2020-318425 33452116

[28] Social Care Institute for Excellence (SCIE). 2022. Hospital discharge and preventing unnecessary hospital admissions (COVID-19). https://www.scie.org.uk/care-providers/coronavirus-covid-19/commissioning/hospital-discharge-admissions.

[29] Zhang J, Garrett S, Sun J. Gastrointestinal symptoms, pathophysiology, and treatment in COVID-19. Genes Dis. 2020; 8(4):385–400. doi: 10.1016/j.gendis.2020.08.013 . 33521210 PMC7836435

[30] Mayo Clinic News Network. Mayo Clinic expert explains gastrointestinal symptoms related to COVID-19. 2020. https://newsnetwork.mayoclinic.org/discussion/mayo-clinic-expert-explains-gastrointestinal-symptoms-related-to-covid-19/.

[31] Haileamlak A. The impact of COVID-19 on health and health systems. Ethiop J Health Sci. 2021 Nov;31(6):1073–4. doi: 10.4314/ejhs.v31i6.1 . 35392335 PMC8968362

[32] Tedla K, Medhin G, Berhe G, Mulugeta A, Berhe N. Delay in treatment initiation and its association with clinical severity and infectiousness among new adult pulmonary tuberculosis patients in Tigray, northern Ethiopia. BMC Infect Dis. 2020; 20(1):456. doi: 10.1186/s12879-020-05191-4 . 32600284 PMC7325053

[33] Imlach F, McKinlay E, Kennedy J, Pledger M, Middleton L, Cumming J, et al. Seeking Healthcare During Lockdown: Challenges, Opportunities and Lessons for the Future. Int J Health Policy Manag. 2021; 1. doi: 10.34172/ijhpm.2021.26 . 33906337 PMC9808356

[34] Topriceanu CC, Wong A, Moon JC, Hughes AD, Bann D, Chaturvedi N, et al. Evaluating access to health and care services during lockdown by the COVID-19 survey in five UK national longitudinal studies. BMJ Open. 2021; 11(3):e045813. doi: 10.1136/bmjopen-2020-045813 . 33737441 PMC7978270

[35] Meyerowitz-Katz G, Bhatt S, Ratmann O, Brauner JM, Flaxman S, Mishra S, et al. Is the cure really worse than the disease? The health impacts of lockdowns during COVID-19. BMJ Glob Health. 2021; 6(8):e006653. doi: 10.1136/bmjgh-2021-006653 . 34281914 PMC8292804

[36] Apisarnthanarak A, Siripraparat C, Apisarnthanarak P, Ullman M, Saengaram P, Leeprechanon N, et al. Patients’ anxiety, fear, and panic related to coronavirus disease 2019 (COVID-19) and confidence in hospital infection control policy in outpatient departments: A survey from four Thai hospitals. Infect Control Hosp Epidemiol. 2021; 42(10):1288–90. doi: 10.1017/ice.2020.1240 . 33023718 PMC7573456

[37] Harvard T. H. Chan School of Public Health. 2021. One in five in U.S. report delayed health care during pandemic. https://www.hsph.harvard.edu/news/hsph-in-the-news/one-in-five-in-u-s-report-delayed-health-care-during-pandemic/.

[38] AAFP. Beware Consequences of Delaying Primary Care in Pandemic. 2020. https://www.aafp.org/news/blogs/freshperspectives/entry/20200625fp-coviddelay.html

[39] Texas A&M Today. 2022. Early In The COVID-19 Pandemic, Many Adults Chose To Delay Or Forego Medical Care. https://today.tamu.edu/2022/03/14/early-in-the-covid-19-pandemic-many-adults-chose-to-delay-or-forego-medical-care/.

[40] MMWR Morb Mortal Wkly Rep. Delay or Avoidance of Medical Care Because of COVID-19–Related Concerns—United States, June 2020. 2020; 69. https://www.cdc.gov/mmwr/volumes/69/wr/mm6936a4.htm. 10.15585/mmwr.mm6936a4 PMC7499838 32915166

[41] World Health Organization. COVID-19 has caused major disruptions and backlogs in health care, new WHO study finds. 2022. https://www.who.int/europe/news/item/20-07-2022-covid-19-has-caused-major-disruptions-and-backlogs-in-health-care—new-who-study-finds.

[42] Singh D, Mathioudakis AG, Higham A. Chronic obstructive pulmonary disease and COVID-19: interrelationships. Curr Opin Pulm Med. 2022; 28(2):76–83. doi: 10.1097/MCP.0000000000000834 . 34690257 PMC8815646

[43] Awatade NT, Wark PAB, Chan ASL, Mamun SAA, Esa NYM, Matsunaga K, et al. The Complex Association between COPD and COVID-19. Journal of Clinical Medicine. 2023; 12(11). doi: 10.3390/jcm12113791 . 37297985 PMC10253799

[44] Terzic CM, Medina-Inojosa BJ. Cardiovascular Complications of COVID-19. Phys Med Rehabil Clin N Am. 2023. . 37419531 10.1016/j.pmr.2023.03.003 PMC10063539

[45] Srithumsuk W, Wangnum K. “New Normal” Home Chemotherapy in Thailand: How the Challenging Roles of Nurses Are Changing? Asia Pac J Oncol Nurs. 2021; 8(3):340–3. doi: 10.4103/apjon.apjon_54_20 . 33850969 PMC8030594

[46] World Health Organization. In Thailand, universal health care eases the impact of diabetes. 2016. https://www.who.int/news-room/feature-stories/detail/in-thailand-universal-health-care-eases-the-impact-of-diabetes.

[47] Reutrakul S, Deerochanawong C. Diabetes in Thailand: Status and Policy. Curr Diab Rep. 2016; 16(3):28. doi: 10.1007/s11892-016-0725-7 . 26894266

[48] Miller L, Alani AH, Avril N, Jingree ML, Atwiine AB, Al Amire K, et al. Adaptation of care for non-communicable diseases during the COVID-19 pandemic: a global case study. BMJ Glob Health. 2022; 7(Suppl 5):e006620. doi: 10.1136/bmjgh-2021-006620 . 35798439 PMC9263348

[49] Spinelli A, Carvello M, Carrano FM, Pasini F, Foppa C, Taffurelli G, et al. Reduced duration of stay after elective colorectal surgery during the peak phase of COVID-19 pandemic: A positive effect of infection risk awareness? Surgery. 2021; 170(2):558–62. doi: 10.1016/j.surg.2020.12.017 . 33714617 PMC7757347

[50] Issac A, Radhakrishnan RV, Vijay V, Stephen S, Krishnan N, Jacob J, et al. An examination of Thailand’s health care system and strategies during the management of the COVID-19 pandemic. J Glob Health. 2021; 11:03002. doi: 10.7189/jogh.11.03002 . 33643614 PMC7897427

[51] AJMC. At-Home Interventions Significantly Cut Readmission Rates in COPD, Study Finds. 2022. https://www.ajmc.com/view/at-home-interventions-significantly-cut-readmission-rates-in-copd-study-finds.

[52] Whitehead M, Taylor-Robinson D, Barr B. Poverty, health, and covid-19. BMJ. 2021; 372:n376. doi: 10.1136/bmj.n376 33579719

[53] Siregar KN, Kurniawan R, BaharuddinNur RJ, Nuridzin DZ, Handayani Y, Retnowati, et al. Potentials of community-based early detection of cardiovascular disease risk during the COVID-19 pandemic. BMC Public Health. 2021; 21:1308. doi: 10.1186/s12889-021-11384-6 . 34217235 PMC8254668

[54] Asch DA, Sheils NE, Islam MN, Chen Y, Werner RM, Buresh J, et al. Variation in US Hospital Mortality Rates for Patients Admitted With COVID-19 During the First 6 Months of the Pandemic. JAMA Intern Med. 2021 Apr 1;181(4):471–8. doi: 10.1001/jamainternmed.2020.8193 . 33351068 PMC7756246

[55] The Centers for Disease Control and Prevention. Provisional Numbers and Rates of Suicide by Month and Demographic Characteristics: United States, 2020. 2022. https://stacks.cdc.gov/view/cdc/120830.

[56] Wasserman D, Iosue M, Wuestefeld A, Carli V. Adaptation of evidence‐based suicide prevention strategies during and after the COVID‐19 pandemic. World Psychiatry. 2020; 19(3):294. doi: 10.1002/wps.20801 . 32931107 PMC7491639

