# Supplementary material for: COVID-19’s impact on hospital stays, mortality, and readmissions for poverty-related diseases, noncommunicable diseases, and injury groups in Thailand
Source: PLoS One. 2024 Sep 11;19(9):e0310090. doi: 10.1371/journal.pone.0310090 (PMC11389936; doi:10.1371/journal.pone.0310090)
Supplement: S1 Table — (DOCX) [file pone.0310090.s001.docx]

**S1 Table Logistic regression analysis of the association between prolonged length of stay, hospital mortality, and readmission of poverty-related diseases group and period of COVID-19 pandemic peak with adjustment for sex, age, and RW**

| **Outcomes of poverty-related diseases group** | **crude OR (95%CI)** | **adjusted OR (95%CI)** | **P value** |
| --- | --- | --- | --- |
| 1. **Prolonged length of stay** |  |  |  |
| 1. **Diarrhea** |  |  |  |
| Period: |  |  |  |
| Pre-COVID-19 pandemic peak | Ref | Ref | < 0.001 |
| During COVID-19 pandemic peak | 1.3 (1.26, 1.33) | 1.16 (1.13, 1.19) |  |
| Post-COVID-19 pandemic peak | 1.34 (1.28, 1.41) | 1.2 (1.15, 1.26) |  |
| 1. **HIV/AIDS** |  |  |  |
| Period: |  |  |  |
| Pre-COVID-19 pandemic peak | Ref | Ref | 0.023 |
| During COVID-19 pandemic peak | 1.16 (1.06, 1.28) | 1.14 (1.04, 1.26) |  |
| Post-COVID-19 pandemic peak | 1.06 (0.88, 1.28) | 1.12 (0.92, 1.37) |  |
| 1. **Lower respiratory infection (LRI)** |  |  |  |
| Period: |  |  |  |
| Pre-COVID-19 pandemic peak | Ref | Ref | <0.001 |
| During COVID-19 pandemic peak | 3.07 (3.03, 3.11) | 3.17 (3.13, 3.22) |  |
| Post-COVID-19 pandemic peak | 1.58 (1.54, 1.62) | 1.21 (1.17, 1.24) |  |
| 1. **Tuberculosis (TB)** |  |  |  |
| Period: |  |  |  |
| Pre-COVID-19 pandemic peak | Ref | Ref | <0.001 |
| During COVID-19 pandemic peak | 1.15 (1.11, 1.18) | 1.04 (1.01, 1.07) |  |
| Post-COVID-19 pandemic peak | 1.02 (0.96, 1.08) | 0.91 (0.85, 0.97) |  |
| 1. **Hospital mortality** |  |  |  |
| 1. **Diarrhea** |  |  |  |
| Period: |  |  |  |
| Pre-COVID-19 pandemic peak | Ref | Ref | 0.003 |
| During COVID-19 pandemic peak | 1.26 (1.18, 1.34) | 1.11 (1.05, 1.19) |  |
| Post-COVID-19 pandemic peak | 1.14 (1.01, 1.29) | 1.03 (0.91, 1.16) |  |
| 1. **HIV/AIDS** |  |  |  |
| Period: |  |  |  |
| Pre-COVID-19 pandemic peak | Ref | Ref | 0.629 |
| During COVID-19 pandemic peak | 1.11 (0.98, 1.24) | 1.04 (0.92, 1.17) |  |
| Post-COVID-19 pandemic peak | 0.96 (0.75, 1.22) | 0.92 (0.72, 1.18) |  |
| 1. **Lower respiratory infection (LRI)** |  |  |  |
| Period: |  |  |  |
| Pre-COVID-19 pandemic peak | Ref | Ref | < 0.001 |
| During COVID-19 pandemic peak | 1.62 (1.6, 1.65) | 1.37 (1.35, 1.39) |  |
| Post-COVID-19 pandemic peak | 1.82 (1.77, 1.86) | 1.22 (1.19, 1.26) |  |
| 1. **Tuberculosis (TB)** |  |  |  |
| Period: |  |  |  |
| Pre-COVID-19 pandemic peak | Ref | Ref | < 0.001 |
| During COVID-19 pandemic peak | 1.23 (1.19, 1.28) | 1.1 (1.02, 1.18) |  |
| Post-COVID-19 pandemic peak | 1.22 (1.14, 1.31) | 1.11 (1.07, 1.16) |  |
| 1. **Readmission** |  |  |  |
| 1. **Diarrhea** |  |  |  |
| Period: |  |  |  |
| Pre-COVID-19 pandemic peak | Ref | Ref | 0.013 |
| During COVID-19 pandemic peak | 0.85 (0.73, 0.99) | 0.88 (0.75, 1.02) |  |
| Post-COVID-19 pandemic peak | 0.64 (0.46, 0.9) | 0.66 (0.47, 0.92) |  |
| 1. **HIV/AIDS** |  |  |  |
| Period: |  |  |  |
| Pre-COVID-19 pandemic peak | Ref | Ref | 0.258 |
| During COVID-19 pandemic peak | 1.21 (0.9, 1.61) | 1.21 (0.91, 1.62) |  |
| Post-COVID-19 pandemic peak | 0.78 (0.41, 1.48) | 0.79 (0.41, 1.51) |  |
| 1. **Lower respiratory infection (LRI)** |  |  |  |
| Period: |  |  |  |
| Pre-COVID-19 pandemic peak | Ref | Ref | < 0.001 |
| During COVID-19 pandemic peak | 0.42 (0.39, 0.44) | 0.43 (0.4, 0.45) |  |
| Post-COVID-19 pandemic peak | 0.57 (0.52, 0.62) | 0.57 (0.52, 0.63) |  |
| 1. **Tuberculosis (TB)** |  |  |  |
| Period: |  |  |  |
| Pre-COVID-19 pandemic peak | Ref | Ref | < 0.001 |
| During COVID-19 pandemic peak | 0.79 (0.72, 0.86) | 0.8 (0.73, 0.87) |  |
| Post-COVID-19 pandemic peak | 0.75 (0.63, 0.9) | 0.76 (0.64, 0.91) |  |
