# Supplementary material for: COVID-19’s impact on hospital stays, mortality, and readmissions for poverty-related diseases, noncommunicable diseases, and injury groups in Thailand
Source: PLoS One. 2024 Sep 11;19(9):e0310090. doi: 10.1371/journal.pone.0310090 (PMC11389936; doi:10.1371/journal.pone.0310090)
Supplement: S2 Table — (DOCX) [file pone.0310090.s002.docx]

**S2 Table Logistic regression analysis of the association between prolonged length of stay, hospital mortality, and readmission of noncommunicable diseases group and period of COVID-19 pandemic peak with adjustment for sex, age, and RW**

| **Outcomes of noncommunicable diseases group** | **crude OR (95%CI)** | **Adjusted OR (95%CI)** | **P value** |
| --- | --- | --- | --- |
| 1. **Prolonged length of stay** |  |  |  |
| 1. **Cancer** |  |  |  |
| Period: |  |  |  |
| Pre-COVID-19 pandemic peak | Ref | Ref | <0.001 |
| During COVID-19 pandemic peak | 0.85 (0.84, 0.86) | 0.8 (0.79, 0.81) |  |
| Post-COVID-19 pandemic peak | 0.75 (0.73, 0.77) | 0.66 (0.65, 0.68) |  |
| 1. **Chronic Obstructive Pulmonary Disease (COPD)** |  |  |  |
| Period: |  |  |  |
| Pre-COVID-19 pandemic peak | Ref | Ref | <0.001 |
| During COVID-19 pandemic peak | 1.2 (1.17, 1.22) | 1.25 (1.22, 1.28) |  |
| Post-COVID-19 pandemic peak | 1.09 (1.05, 1.14) | 1.13 (1.08, 1.17) |  |
| 1. **Cardiovascular disease (CVD)** |  |  |  |
| Period: |  |  |  |
| Pre-COVID-19 pandemic peak | Ref | Ref | <0.001 |
| During COVID-19 pandemic peak | 0.95 (0.92, 0.99) | 0.91 (0.87, 0.94) |  |
| Post-COVID-19 pandemic peak | 0.5 (0.46, 0.55) | 0.5 (0.45, 0.55) |  |
| 1. **Diabetes mellitus (DM)** |  |  |  |
| Period: |  |  |  |
| Pre-COVID-19 pandemic peak | Ref | Ref | 0.158 |
| During COVID-19 pandemic peak | 0.95 (0.88, 1.04) | 0.94 (0.87, 1.03) |  |
| Post-COVID-19 pandemic peak | 0.88 (0.75, 1.04) | 0.87 (0.74, 1.03) |  |
| 1. **Hospital mortality** |  |  |  |
| 1. **Cancer** |  |  |  |
| Period: |  |  |  |
| Pre-COVID-19 pandemic peak | Ref | Ref | <0.001 |
| During COVID-19 pandemic peak | 0.78 (0.77, 0.8) | 0.77 (0.76, 0.78) |  |
| Post-COVID-19 pandemic peak | 0.51 (0.49, 0.53) | 0.5 (0.48, 0.51) |  |
| 1. **Chronic Obstructive Pulmonary Disease (COPD)** |  |  |  |
| Period: |  |  |  |
| Pre-COVID-19 pandemic peak | Ref | Ref | <0.001 |
| During COVID-19 pandemic peak | 0.81 (0.78, 0.84) | 0.75 (0.72, 0.78) |  |
| Post-COVID-19 pandemic peak | 0.65 (0.6, 0.7) | 0.57 (0.53, 0.62) |  |
| 1. **Cardiovascular disease (CVD)** |  |  |  |
| Period: |  |  |  |
| Pre-COVID-19 pandemic peak | Ref | Ref | < 0.001 |
| During COVID-19 pandemic peak | 0.97 (0.96, 0.98) | 0.95 (0.94, 0.96) |  |
| Post-COVID-19 pandemic peak | 0.85 (0.82, 0.87) | 0.84 (0.82, 0.87) |  |
| 1. **Diabetes mellitus (DM)** |  |  |  |
| Period: |  |  |  |
| Pre-COVID-19 pandemic peak | Ref | Ref | <0.001 |
| During COVID-19 pandemic peak | 1.65 (1.23, 2.21) | 1.61 (1.2,2. 15) |  |
| Post-COVID-19 pandemic peak | 2.36 (1.47, 3.76) | 2.31 (1.44, 3.7) |  |
| 1. **Hospital readmission** |  |  |  |
| 1. **Cancer** |  |  |  |
| Period: |  |  |  |
| Pre-COVID-19 pandemic peak | Ref | Ref | < 0.001 |
| During COVID-19 pandemic peak | 0.7 (0.69, 0.72) | 0.71 (0.69, 0.73) |  |
| Post-COVID-19 pandemic peak | 0.56 (0.53, 0.59) | 0.57 (0.54, 0.6) |  |
| 1. **Chronic Obstructive Pulmonary Disease (COPD)** |  |  |  |
| Period: |  |  |  |
| Pre-COVID-19 pandemic peak | Ref | Ref | < 0.001 |
| During COVID-19 pandemic peak | 0.6 (0.58, 0.62) | 0.6 (0.58, 0.62) |  |
| Post-COVID-19 pandemic peak | 0.51 (0.47, 0.54) | 0.5 (0.47, 0.53) |  |
| 1. **Cardiovascular disease (CVD)** |  |  |  |
| Period: |  |  |  |
| Pre-COVID-19 pandemic peak | Ref | Ref | < 0.001 |
| During COVID-19 pandemic peak | 0.84 (0.81, 0.88) | 0.84 (0.81, 0.88) |  |
| Post-COVID-19 pandemic peak | 0.71 (0.66, 0.76) | 0.71 (0.66, 0.76) |  |
| 1. **Diabetes mellitus (DM)** |  |  |  |
| Period: |  |  |  |
| Pre-COVID-19 pandemic peak | Ref | Ref | 0.594 |
| During COVID-19 pandemic peak | 0.93 (0.45, 1.92) | 0.92 (0.45, 1.91) |  |
| Post-COVID-19 pandemic peak | 0.41 (0.05, 3.06) | 0.4 (0.05, 3.02) |  |
