# Supplementary material for: COVID-19’s impact on hospital stays, mortality, and readmissions for poverty-related diseases, noncommunicable diseases, and injury groups in Thailand
Source: PLoS One. 2024 Sep 11;19(9):e0310090. doi: 10.1371/journal.pone.0310090 (PMC11389936; doi:10.1371/journal.pone.0310090)
Supplement: S3 Table — (DOCX) [file pone.0310090.s003.docx]

**S3 Table Logistic regression analysis of the association between prolonged length of stay, hospital mortality, and readmission of injuries group and period of COVID-19 pandemic peak with adjustment for sex, age, and RW**

| **Outcomes of injuries group** | **crude OR (95%CI)** | **adjusted OR (95%CI)** | **P value** |
| --- | --- | --- | --- |
| 1. **Prolonged length of stay** |  |  |  |
| 1. **Road traffic accident** |  |  |  |
| Period: |  |  |  |
| Pre-COVID-19 pandemic peak | Ref | Ref | <0.001 |
| During COVID-19 pandemic peak | 0.9 (0.89, 0.91) | 0.88 (0.86, 0.89) |  |
| Post-COVID-19 pandemic peak | 0.84 (0.82, 0.87) | 0.81 (0.79, 0.83) |  |
| 1. **Suicide** |  |  |  |
| Period: |  |  |  |
| Pre-COVID-19 pandemic peak | Ref | Ref | 0.612 |
| During COVID-19 pandemic peak | 1 (0.94, 1.05) | 1.03 (0.97, 1.09) |  |
| Post-COVID-19 pandemic peak | 0.8733  (0.7926, 0.9622) | 0.9933 (0.8945, 1.1031) |  |
| 1. **Hospital mortality** |  |  |  |
| 1. **Road traffic accident** |  |  |  |
| Period: |  |  |  |
| Pre-COVID-19 pandemic peak | Ref | Ref | 0.423 |
| During COVID-19 pandemic peak | 0.98 (0.95, 1.02) | 0.98 (0.95, 1.01) |  |
| Post-COVID-19 pandemic peak | 0.982  (0.9194, 1.0488) | 1.0078  (0.9426, 1.0775) |  |
| 1. **Suicide** |  |  |  |
| Period: |  |  |  |
| Pre-COVID-19 pandemic peak | Ref | Ref | < 0.001 |
| During COVID-19 pandemic peak | 0.69 (0.64, 0.74) | 0.75 (0.69, 0.81) |  |
| Post-COVID-19 pandemic peak | 0.41 (0.35, 0.48) | 0.53 (0.45, 0.63) |  |
| 1. **Hospital readmission** |  |  |  |
| 1. **Road traffic accident** |  |  |  |
| Period: |  |  |  |
| Pre-COVID-19 pandemic peak | Ref | Ref | 0.143 |
| During COVID-19 pandemic peak | 0.91 (0.79, 1.05) | 0.91 (0.79, 1.05) |  |
| Post-COVID-19 pandemic peak | 1.16 (0.9, 1.49) | 1.16 (0.9, 1.49) |  |
| 1. **Suicide** |  |  |  |
| Period: |  |  |  |
| Pre-COVID-19 pandemic peak | Ref | Ref | 0.025 |
| During COVID-19 pandemic peak | 0.69 (0.46, 1.03) | 0.72 (0.48, 1.08) |  |
| Post-COVID-19 pandemic peak | 0.31 (0.11, 0.84) | 0.34 (0.12, 0.92) |  |
