# Supplementary material for: COVID-19’s impact on hospital stays, mortality, and readmissions for poverty-related diseases, noncommunicable diseases, and injury groups in Thailand
Source: PLoS One. 2024 Sep 11;19(9):e0310090. doi: 10.1371/journal.pone.0310090 (PMC11389936; doi:10.1371/journal.pone.0310090)
Supplement: Attachment — Submitted filename: 20 Juni 2024 Response to reviewers.docx [file pone.0310090.s004.docx]

1. **Additional requirement 1**

Please ensure that your manuscript meets PLOS ONE's style requirements, including those for file naming

**Response**

We have already re-checked and revised PLOS ONE's style requirements.

2. **Additional requirement 2**

Thank you for stating the following financial disclosure:
"Research reported in this publication was supported by Fogarty International Center and the National Institute of Allergy and Infectious Diseases of the National Institutes of Health on the project “TB/MDR-TB Research Capacity Building in low and middle-income countries in Southeast Asia” under Awarded Number D43TW009522."

Please state what role the funders took in the study.  If the funders had no role, please state: "The funders had no role in study design, data collection and analysis, decision to publish, or preparation of the manuscript." 
If this statement is not correct you must amend it as needed. 
Please include this amended Role of Funder statement in your cover letter; we will change the online submission form on your behalf.

**Response**

- We have added the statement: "The funders had no role in study design, data collection and analysis, decision to publish, or preparation of the manuscript." in the PLOS ONE submission system.
- We added this amended Role of Funder statement in the cover letter (attached).

3. **Additional requirement 3**

Thank you for stating the following in the Acknowledgments Section of your manuscript: 
"Thanks to the Fogarty International Center and the National Institute of Allergy and Infectious Diseases of the National Institutes of Health on the project “TB/MDR-TB Research Capacity Building in low and middle-income countries in Southeast Asia” under Awarded Number D43TW009522, for the support."

We note that you have provided funding information that is not currently declared in your Funding Statement. However, funding information should not appear in the Acknowledgments section or other areas of your manuscript. We will only publish funding information present in the Funding Statement section of the online submission form. 
Please remove any funding-related text from the manuscript and let us know how you would like to update your Funding Statement. Currently, your Funding Statement reads as follows: 
"Research reported in this publication was supported by Fogarty International Center and the National Institute of Allergy and Infectious Diseases of the National Institutes of Health on the project “TB/MDR-TB Research Capacity Building in low and middle-income countries in Southeast Asia” under Awarded Number D43TW009522."

Please include your amended statements within your cover letter; we will change the online submission form on your behalf.

**Response**

We revised as suggested in the acknowledgments as follows:

This study was a part of a a post-doctoral trainee of Satiti Palupi at the Department of Epidemiology, Faculty of Medicine, Prince of Songkla University, Hat Yai, Songkhla, Thailand. We also thank the Thai Health Information Portal **(**THIP) for providing the dataset.

Also, we added amended statements within the cover letter (attached).

4. **Additional requirement 4**

We note that you have indicated that there are restrictions to data sharing for this study. For studies involving human research participant data or other sensitive data, we encourage authors to share de-identified or anonymized data. However, when data cannot be publicly shared for ethical reasons, we allow authors to make their data sets available upon request. For information on unacceptable data access restrictions, please see <http://journals.plos.org/plosone/s/data-availability#loc-unacceptable-data-access-restrictions>.

Before we proceed with your manuscript, please address the following prompts:

a) If there are ethical or legal restrictions on sharing a de-identified data set, please explain them in detail (e.g., data contain potentially identifying or sensitive patient information, data are owned by a third-party organization, etc.) and who has imposed them (e.g., a Research Ethics Committee or Institutional Review Board, etc.). Please also provide contact information for a data access committee, ethics committee, or other institutional body to which data requests may be sent.

b) If there are no restrictions, please upload the minimal anonymized data set necessary to replicate your study findings to a stable, public repository and provide us with the relevant URLs, DOIs, or accession numbers. Please see <http://www.bmj.com/content/340/bmj.c181.long> for guidelines on how to de-identify and prepare clinical data for publication. For a list of recommended repositories, please see <https://journals.plos.org/plosone/s/recommended-repositories>. You also have the option of uploading the data as Supporting Information files, but we would recommend depositing data directly to a data repository if possible.

Please update your Data Availability statement in the submission form accordingly.

**Response**

The study was conducted in accordance with the Declaration of Helsinki, and approved by the Human Research Ethics Committee, Prince of Songkla University (REC number 66-485-18-1). Patient data were encrypted for personalized anonymization according to the Thai Personal Data Protection Act 2019, Thailand (PDPA). All information in the analysis is available in the Supplementary Files. The full data sets of all patients could not be shared according to PDPA.

Data is available to request following the processes described in "<https://thip.nbt.or.th/documents/THIP-Manual.pdf> " in which detailed procedure of data request and contact information were included.

5. **Additional requirement 5**

Please include your full ethics statement in the ‘Methods’ section of your manuscript file. In your statement, please include the full name of the IRB or ethics committee who approved or waived your study, as well as whether or not you obtained informed written or verbal consent. If consent was waived for your study, please include this information in your statement as well.

**Response**

As suggested in the Materials and Methods section (attached), we have added the information above.

**Before revision**

In this study, THIP data was accessed between September 5, 2023, and December 16, 2023. The Institutional Ethics Committee of the Faculty of Medicine at Prince of Songkla University in Hat Yai, Thailand, granted ethical approval under reference number REC 66-485-18-1.

**After revision**

In this study, THIP data was accessed between September 5, 2023, and December 16, 2023. The Institutional Ethics Committee of the Faculty of Medicine at Prince of Songkla University in Hat Yai, Thailand, granted ethical approval under reference number REC 66-485-18-1. Prof. Boonsin Tangtrakulwanich, Chairman of the Human Research Ethics Committee, Faculty of Medicine, Prince of Songkla University, approved the study.

6. **Additional requirement 6**

 Please modify the title to be more precise and attractive, i.e., include some information about diseases in focus and which hospital quality's parameter were measured and reported.

**Response**

We changed the title as suggested.

**Before revision**

Quality of hospital care for WHO's three disease groups pre-, during, and post-COVID-19 pandemic peak in Thailand

**After revision**

COVID-19's Impact on Hospital Stays, Mortality, and Readmissions for Poverty-Related Diseases, Noncommunicable Diseases, and Injury Groups in Thailand

Feedback to author

Reviewer 1

The study is not novel, and similar papers have been published before.

Response

Thank you for your valuable feedback.

We acknowledge that previous studies have examined the impact of the COVID-19 pandemic on healthcare utilization and outcomes. However, our study makes a distinct contribution by focusing specifically on the Thai healthcare context and analyzing trends in hospital care quality for three distinct disease groups (poverty-related, noncommunicable, and injuries) across the pre-, during-, and post-pandemic peak periods. We believe that these findings have important implications for healthcare planning and resource allocation in Thailand, especially as the country continues to recover from the pandemic.

**Reviewer 2**

Reviewer #2: The article titled "Quality of Hospital Care for WHO's Three Disease Groups Pre-, During, and Post-COVID-19 Pandemic Peak in Thailand," submitted to the scientific journal Plos One, delves into the critical examination of hospital care quality across different disease groups during various phases of the COVID-19 pandemic. Through analyzing trends in hospital admission data from the Thai Health Information Portal (THIP) database, the study aims to shed light on how the pandemic has affected the quality of care for poverty-related, noncommunicable, and injury-related diseases in Thailand.

Positive Points:
Relevance and Timeliness: The study addresses a timely and significant issue, providing insights into how the COVID-19 pandemic has impacted the quality of hospital care across different disease groups. This research is particularly valuable given the ongoing global healthcare crisis and the need to understand its implications on healthcare delivery systems.

Comprehensive Methodology: The utilization of existing hospital admission data from the THIP database covering a substantial period from 2017 to 2022 lends credibility to the study. Categorizing disease groups using the International Classification of Diseases (ICD)-10 and analyzing outcomes such as prolonged length of stay (LOS), hospital mortality, and readmission pre-, during, and post-COVID-19 pandemic peak offers a comprehensive view of the situation.

Insightful Findings: The study's findings provide valuable insights into the impact of the pandemic on different disease groups. By examining metrics such as prolonged LOS, hospital mortality rates, and readmissions, the research highlights the varying degrees of impact across poverty-related, noncommunicable, and injury-related diseases, contributing to a deeper understanding of healthcare dynamics during the pandemic.

Opportunities for Improvement:

Clarity in Data Presentation: While the study presents extensive data on outcomes across disease groups, there is a need for clearer presentation and interpretation of results. Providing visual aids such as tables or graphs could enhance the readability and accessibility of the findings for readers.

Discussion of Methodological Limitations: The article lacks a comprehensive discussion of the limitations inherent in the methodology employed. Addressing potential biases, data limitations, and other methodological constraints would strengthen the study's validity and assist readers in interpreting the results accurately.

Contextual Analysis: While the study identifies the impact of COVID-19 on hospital care quality, it could benefit from a deeper contextual analysis of the underlying factors influencing these trends. Exploring socioeconomic determinants, healthcare infrastructure, and public health interventions could enrich the interpretation of findings and provide actionable insights for policymakers and healthcare practitioners.

Future Research Directions: The article would benefit from a section dedicated to suggesting avenues for future research. Identifying gaps in knowledge, proposing alternative methodologies, or recommending areas for further investigation would enhance the scholarly contribution of the study and guide future research efforts in this area.

In conclusion, the article "Quality of Hospital Care for WHO's Three Disease Groups Pre-, During, and Post-COVID-19 Pandemic Peak in Thailand" presents a valuable contribution to understanding healthcare quality dynamics during the COVID-19 pandemic. While the study demonstrates strengths in relevance, methodology, and findings, there are opportunities for improvement in data presentation, discussion of methodological limitations, contextual analysis, and identification of future research directions. Addressing these areas would enhance the overall quality and impact of the research.

**Response**

Thank you for your insightful comments and positive feedback on our manuscript. We have already added sentences and revised them for improvement as suggested.

| No | Feedback from reviewer | Response |
| --- | --- | --- |
| 1 | **Clarity in Data Presentation:**  While the study presents extensive data on outcomes across disease groups, there is a need for clearer presentation and interpretation of results. Providing visual aids such as tables or graphs could enhance the readability and accessibility of the findings for readers. | For Clarity in Data Presentation, we have enhanced the presentation of results by incorporating tables and graphs to provide a clearer and more accessible overview of the findings. Specifically, we have added a table summarizing the direction of effects and consistent findings among the three major disease groups (Table 7). Additionally, we have included supplementary tables (S1, S2, and S3 Tables) presenting the results of logistic regression analyses, further clarifying the statistical associations observed. |
| 2 | **Discussion of Methodological Limitations:**  The article lacks a comprehensive discussion of the limitations inherent in the methodology employed. Addressing potential biases, data limitations, and other methodological constraints would strengthen the study's validity and assist readers in interpreting the results accurately. | For the Discussion of Methodological Limitations, we already revised the Discussion section in paragraph 15  **Before revision**  The limitations of this study were that it did not conclude all the diseases are there hospitalized, and the data utilized may not have comprehensively captured the broader health outcomes of the entire population, as the study primarily relies on hospital-based metrics and lack of information level of complication. That limitation emphasizes the need for future research to adopt a more holistic approach, incorporating community-level health indicators and qualitative assessments to better understand the comprehensive implications of healthcare changes during and post-COVID-19 pandemic peak.  **After revision:**  This study has several limitations. First, it relied on hospital admission data, which might not fully capture the broader health outcomes of the entire population. Second, the data lacks information on disease severity, which could be a potential confounder in the analysis. Third, the use of relative weight (RW) as a surrogate measure of disease severity has its limitations, as it may not fully reflect the complexity and heterogeneity of individual cases. Finally, the study is observational. These limitations emphasize the need for future research to adopt a more holistic approach, incorporating community-level health indicators and qualitative assessments to better understand the comprehensive implications of healthcare changes during and post-COVID-19 pandemic peak. |
| 3 | **Contextual Analysis:**  While the study identifies the impact of COVID-19 on hospital care quality, it could benefit from a deeper contextual analysis of the underlying factors influencing these trends. Exploring socioeconomic determinants, healthcare infrastructure, and public health interventions could enrich the interpretation of findings and provide actionable insights for policymakers and healthcare practitioners. | Our manuscript processes data from the THIP database or patients being hospitalized in Thailand. The data we used did not include information about determinants of socioeconomic status such as income, education, or occupation. However, this manuscript could provide a glimpse of information regarding SES from the group of diseases related to poverty-related diseases.  If we discuss healthcare infrastructure and public health intervention, this is a limitation of this manuscript. |
| 4. | **Future Research Directions:**  The article would benefit from a section dedicated to suggesting avenues for future research. Identifying gaps in knowledge, proposing alternative methodologies, or recommending areas for further investigation would enhance the scholarly contribution of the study and guide future research efforts in this area. | We revised and added sentences it as suggested  **Before revision**  The limitations of this study were that it did not conclude all the diseases are there hospitalized, and the data utilized may not have comprehensively captured the broader health outcomes of the entire population, as the study primarily relies on hospital-based metrics and lack of information level of complication. That limitation emphasizes the need for future research to adopt a more holistic approach, incorporating community-level health indicators and qualitative assessments to better understand the comprehensive implications of healthcare changes during and post-COVID-19 pandemic peak.  **After revision**  This study has several limitations. First, it relies on hospital admission data, which may not fully capture the broader health outcomes of the entire population. Second, the data lacks information on disease severity, which could be a potential confounder in the analysis. Third, the use of relative weight (RW) as a surrogate measure of disease severity has its limitations, as it may not fully reflect the complexity and heterogeneity of individual cases. Finally, the study is observational. That limitation emphasizes the need for future research to adopt a more holistic approach to understanding the comprehensive implications of healthcare changes during and post-COVID-19, incorporating community-level health indicators and qualitative assessments alongside quantitative data. This research should investigate the long-term impact of the pandemic on hospital care quality, examine the effects on specific subpopulations, and explore health disparities, the role of healthcare infrastructure, and the effectiveness of public health interventions. Comparative studies across different countries could provide valuable insights for global pandemic preparedness and response. |

**Reviewer 3**

The manuscript described hospital quality variables in different periods of COVID-19 pandemic.
I have some comments as follows:
1. Please modify the title to be more precise and attractive, i.e., include some information about diseases in focus and which hospital quality's parameter were measured and reported.

2. Introduction:

a. Please include more relevant references, esp. profound healthcare disruption from the pandemic (ref.1), and ref. 2. For example, information from CDC or WHO.
b. Moreover, there were a lot of published articles about the impact of the pandemics on healthcare quality, including those variables focused on this manuscript. Please elaborate on how would this manuscript benefit the scientific society?

3. Method:

a. Please use weighted data to analyse the regression, since the sample size of post pandemic period was much smaller than other period.
b. What are the effect size of each primary outcome.

4. Results:

a. Please add more information on demographic data in each period. For example, numbers of poverty-related/non-communicable cases in each period.
b. Regression table: it was interesting that there were significance differences among periods; however, they did not show which period differ from which. From the analytic results presented, it can be that pre-pandemic period gave differences in outcomes than post-pandemic period without the relation to pandemic period.
c. 'DM' and 'CVD' are covariate, having both of them in the model can induce bias.

5. Discussion:

a. Some analysis demonstrated similar levels and trends in during and post-COVID-19 periods. The authors discussed only the differences during the pandemic, and seemed to neglect the post pandemic's data.
b. Second page of discussion, line 12-14 -> please reference the statement

11 Lockdowns may limit people's ability
12 to seek timely healthcare for various conditions, leading to more severe presentations when they
13 eventually seek treatment. More severe cases often require longer hospital stays or hospital
14 mortality.

c. Second page of discussion,line 26; indicating potential challenges in managing these conditions -> please elaborate on this statement.
d. Second page of discussion,line 28-29:' The findings for CVD and DM exhibited mixed directions, highlighting the complexity of the pandemic's influence on hospital care across various health issues.' since these diseases can be covariate , it is possible that they manifested similar issues.

e. Please revise Academic written English language, particularly in discussion part'
f. Please add results' implications into the last part of the discussion or in conclusion.

**Response**

| **No** | **Feedback from reviewer** | **Response** |
| --- | --- | --- |
| 1 | Please modify the title to be more precise and attractive, i.e., include some information about diseases in focus and which hospital quality's parameter were measured and reported. | We changed the title as suggested.  **Before revision:**  Quality of Hospital Care for WHO's Three Disease Groups Pre-, During, and Post-COVID-19 Pandemic Peak in Thailand.  **After revision:**  COVID-19's Impact on Hospital Stays, Mortality, and Readmissions for Poverty-Related Diseases, Noncommunicable Diseases, and Injury Groups in Thailand. |
| 2. | Introduction:   a. Please include more relevant references, esp. profound healthcare disruption from the pandemic (ref.1), and ref. 2. For example, information from CDC or WHO. b. Moreover, there were a lot of published articles about the impact of the pandemics on healthcare quality, including those variables focused on this manuscript. Please elaborate on how would this manuscript benefit the scientific society? | a. We have already revised it and added sentences with more relevant references, as suggested in the Introduction section in paragraph 1.   **Before revision:**  On March 11, 2020, the World Health Organization (WHO) officially declared the COVID-19 pandemic as a worldwide crisis. This unprecedented event significantly affected various aspects of human life, particularly in the realm of healthcare. Essential health services were disrupted in 90% of countries across the world, leading to profound implications for the quality of healthcare people received during the pandemic (1). The COVID-19 pandemic has undoubtedly affected the quality of healthcare services globally. During the pandemic's peak, non-COVID-19 patients experienced a decreased quality of hospital care or outcomes (2).  **After Revision:**  On March 11, 2020, the World Health Organization (WHO) officially declared the COVID-19 pandemic as a worldwide crisis. This unprecedented event significantly affected various aspects of human life, particularly in the realm of healthcare. Essential health services were disrupted in 90% of countries across the world, with disruptions including resource constraints, reductions in elective procedures, and delays in essential care, as highlighted by the WHO (1–3). These disruptions had profound implications for the quality of healthcare people received during the pandemic, as non-COVID-19 patients experienced a decreased quality of hospital care or outcomes (4).  We also revised the Reference section, added references from WHO  **Before revision**   1. Huggins A, Husaini M, Wang F, Waken R, Epstein AM, Orav EJ, et al. Care Disruption During COVID-19: a National Survey of Hospital Leaders. J Gen Intern Med. 2023 Apr;38(5): 1232–8. <https://doi.org/10.1007/s11606-022-08002> 2. Sabetkish N, Rahmani A. The overall impact of COVID-19 on healthcare during the pandemic: A multidisciplinary point of view. Health Sci Rep. 2021 Dec;4(4): e386. <https://doi.org/10.1002/hsr2.386>.   **After revision**   1. Huggins A, Husaini M, Wang F, Waken R, Epstein AM, Orav EJ, et al. Care Disruption During COVID-19: a National Survey of Hospital Leaders. J Gen Intern Med. 2023; 38(5):1232–8. <https://doi.org/10.1007%2Fs11606-022-08002-5> PMID: [36650332](https://pubmed.ncbi.nlm.nih.gov/36650332). 2. World Health Organization. COVID-19 continues to disrupt essential health services in 90% of countries. 2021. <https://www.who.int/news/item/23-04-2021-covid-19-continues-to-disrupt-essential-health-services-in-90-of-countries>. 3. World Health Organization. Essential health services face continued disruption during COVID-19 pandemic. 2022.   <https://www.who.int/news/item/07-02-2022-essential-health-services-face-continued-disruption-during-covid-19-pandemic>.   4. Sabetkish N, Rahmani A. The overall impact of COVID-19 on healthcare during the pandemic: A multidisciplinary point of view. Health Sci Rep. 2021; 4(4). <https://doi.org/10.1002/hsr2.386>. b. We added sentences as suggested in the Discussion section in paragraph 15.   **Before revision:**  These findings provide a nuanced understanding of the multifaceted impact of the pandemic on hospital care quality across different disease categories in the Thai healthcare system, offering valuable insights for future healthcare planning and improvements.  The limitations of this study were that it did not conclude all the diseases are there hospitalized, and the data utilized may not have comprehensively captured the broader health outcomes of the entire population, as the study primarily relies on hospital-based metrics and lack of information level of complication. That limitation emphasizes the need for future research to adopt a more holistic approach, incorporating community-level health indicators and qualitative assessments to better understand the comprehensive implications of healthcare changes during and post-COVID-19 pandemic peak.  **After revision**  These findings provide a nuanced understanding of the pandemic's multifaceted impact on hospital care quality across different disease categories in the Thai healthcare system, offering valuable insights for future healthcare planning and improvements.  Furthermore, using relative weight (RW) as a surrogate measure of disease severity provides a novel perspective on the pandemic's economic impact on healthcare resource utilization and costs, adding a unique dimension to the existing literature.  This study has several limitations. First, it relies on hospital admission data, which may not fully capture the broader health outcomes of the entire population. Second, the data lacks information on disease severity, which could be a potential confounder in the analysis. Third, the use of relative weight (RW) as a surrogate measure of disease severity has its limitations, as it may not fully reflect the complexity and heterogeneity of individual cases. Finally, the study is observational in nature. That limitation emphasizes the need for future research to adopt a more holistic approach to understanding the comprehensive implications of healthcare changes during and post-COVID-19, incorporating community-level health indicators and qualitative assessments alongside quantitative data. This research should investigate the long-term impact of the pandemic on hospital care quality, examine the effects on specific subpopulations, and explore health disparities, the role of healthcare infrastructure, and the effectiveness of public health interventions. Comparative studies across different countries could provide valuable insights for global pandemic preparedness and response. |
| 3. | Method:   a. Please use weighted data to analyse the regression, since the sample size of post pandemic period was much smaller than other period. b. What are the effect size of each primary outcome | a. Thanks for this interesting suggestion. Our data are from the whole population. There is no sampling process. Weighted regression is used for subjects from samples with different sampling fractions, which is not our case. We agree that there is a difference in the number of denominators in different periods of COVID-19 phases.  Different phases are actually a variable of the COVID-19 period. This is from nature just like one will have differences in sexes and age groups in the population. We hope you agree with this humble explanation. b. We used estimates of adjusted odds ratio as the effect size of each primary outcome. |
| 4. | Results:   a. Please add more information on demographic data in each period. For example, numbers of poverty-related/non-communicable cases in each period. b. Regression table: it was interesting that there were significance differences among periods; however, they did not show which period differ from which. From the analytic results presented, it can be that pre-pandemic period gave differences in outcomes than post-pandemic period without the relation to pandemic period. c. 'DM' and 'CVD' are covariate, having both of them in the model can induce bias. | a. We have already included it in Table 1. b. In the regression table, the pre-pandemic period was used as the reference to compare the during-pandemic period and the post-pandemic period, respectively. The significant difference was interpreted as the difference between the pre-pandemic period and the during-pandemic period as well as between the pre-pandemic period and the post-pandemic period. c. We analyzed each covariate separately. We included it in the discussion. |
| 5. | Discussion:   a. Some analysis demonstrated similar levels and trends in during and post-COVID-19 periods. b. Second page of discussion, line 12-14 -> please reference the statement   11 Lockdowns may limit people's ability 12 to seek timely healthcare for various conditions, leading to more severe presentations when they 13 eventually seek treatment. More severe cases often require longer hospital stays or hospital 14 mortality.   c. Second page of discussion,line 26; indicating potential challenges in managing these conditions -> please elaborate on this statement. d. Second page of discussion, line 28-29:' The findings for CVD and DM exhibited mixed directions, highlighting the complexity of the pandemic's influence on hospital care across various health issues.' since these diseases can be covariate , it is possible that they manifested similar issues. e. Please revise Academic written English language, particularly in discussion part'. f. Please add results' implications into the last part of the discussion or in conclusion. | a. The discussion points summarized disease outcomes during, and post-pandemic periods compared to the pre-pandemic period. We revised the sentences to highlight the impacts of both during and post-pandemic on disease outcomes. b. We revised and added reference for sentence as suggestion in the Discussion section paragraph 6 pages 2 line 12-13 and added new paragraph below it as paragraph 7.   **Before revision**  Respiratory distress and gastrointestinal symptoms are common in many infectious diseases such as COVID-19, influenza, pneumonia, and even TB (27,28). The rule out COVID-19 transmission in the pandemic period, the hospitalization period needed to be extended, which led to the lengthening of LOS. Patients might have prolonged delays in arriving at the hospital to get service due to worrying about getting a COVID-19 infection from the hospital and inconvenience in transportation and the referral system. These delays would increase the level of clinical complication, which leads to extended LOS. The change of pattern in healthcare systems during the pandemic could result in delayed access to medical care, leading to more severe presentations of these conditions when patients finally seek treatment (29). Lockdowns may limit people's ability to seek timely healthcare for various conditions, leading to more severe presentations when they eventually seek treatment. More severe cases often require longer hospital stays or hospital mortality.  **After revision**  Respiratory distress and gastrointestinal symptoms are common in many infectious diseases such as COVID-19, influenza, pneumonia, and even TB (29,30). The rule out COVID-19 transmission in the pandemic period, the hospitalization period needed to be extended, which led to the lengthening of LOS. Patients might have prolonged delays in arriving at the hospital to get service due to worrying about getting a COVID-19 infection from the hospital and inconvenience in transportation and the referral system. These delays would increase the level of clinical complication, which leads to extended LOS. The change of pattern in healthcare systems during the pandemic could result in delayed access to medical care, leading to more severe presentations of these conditions when patients finally seek treatment (31).  Lockdowns may limit people's ability to seek timely healthcare for various conditions, leading to more severe presentations when they eventually seek treatment. In addition, during the pandemic, delayed diagnosis of new cases often led to severe disease complications (32). These new cases of complications are also probably accumulated in the post-pandemic period, particularly for poverty-related diseases such as TB, respiratory diseases, and HIV. Although delayed diagnosis of new cases can also occur in COPD and cancer patients, the slow progression of these new diseases means they are less likely to present as severe cases in the post-pandemic period. More severe cases often require longer hospital stays or hospital mortality. More severe cases often require longer hospital stays or hospital mortality (33–35).  **Revision in the Reference section:**  32. Tedla K, Medhin G, Berhe G, Mulugeta A, Berhe N. Delay in treatment initiation and its association with clinical severity and infectiousness among new adult pulmonary tuberculosis patients in Tigray, northern Ethiopia. BMC Infect Dis. 2020; 20(1):456. <https://doi.org/10.1186/s12879-020-05191-4> PMID: 32600284.  33. Imlach F, McKinlay E, Kennedy J, Pledger M, Middleton L, Cumming J, et al. Seeking Healthcare During Lockdown: Challenges, Opportunities and Lessons for the Future. Int J Health Policy Manag. 2021; 1. <https://doi.org/10.34172/ijhpm.2021.26> PMID: 33906337.  34. Topriceanu CC, Wong A, Moon JC, Hughes AD, Bann D, Chaturvedi N, et al. Evaluating access to health and care services during lockdown by the COVID-19 survey in five UK national longitudinal studies. BMJ Open. 2021; 11(3): e045813. <https://doi.org/10.1136/bmjopen-2020-045813> PMID: 33737441.  34. Meyerowitz-Katz G, Bhatt S, Ratmann O, Brauner JM, Flaxman S, Mishra S, et al. Is the cure really worse than the disease? The health impacts of lockdowns during COVID-19. BMJ Glob Health. 2021; 6(8): e006653. <https://doi.org/10.1136%2Fbmjgh-2021-006653> PMID: [34281914](https://pubmed.ncbi.nlm.nih.gov/34281914).  c. We revised it as the suggestion in the discussion section paragraph 9, pages 2, line 26.  **Before revision**  Prolonged LOS consistently increased during and post-COVID-19 pandemic peak for COPD because COPD shares similarities with COVID-19 (36,37), indicating potential challenges in managing these conditions.  **After revision**  Prolonged LOS consistently increased during and post-COVID-19 pandemic peak for COPD because COPD patients were at increased risk of severe COVID-19 outcomes and vice versa. COVID-19 exacerbates the severity of COPD patient's outcomes (36,37), indicating potential challenges in providing treatment and care at the hospital.   g. We already revised as suggestion in the Discussion section paragraph 9, pages 2, lines 28-19   **Before revision**  The findings for CVD and DM exhibited mixed directions, highlighting the complexity of the pandemic's influence on hospital care across various health issues. This result related to the previous study in the USA that cardiovascular complications are a common manifestation of acute and post-acute COVID-19 infection  **After revision**  The findings for CVD and DM exhibited mixed directions, highlighting the complexity of the pandemic's influence on hospital care across various health issues. In addition, since these diseases can be comorbid, it is possible that they manifest similar issues. This result related to the previous study in the USA that cardiovascular complications are a common manifestation of acute and post-acute COVID-19 infection (38)   d. We revised sentences in Discussion section to make Academic language in the paragraph 4 and paragraph 9.   Paragraph 4  **Before revision**  Effects on hospital readmission were quite different from that on length of stay. In general, readmission is partially affected too early discharge (26), when the final diagnosis and/or treatment were not reached. COVID-19 pandemic decreased hospital general case load due to lock down process as well as people were generally scared of contract the infection. Reduction of general case load thus could reduce the needs for early discharge of the patients to reduce hospital congestion reduction of early discharge, thus reduce the chance of readmission.  **After revision**  Effects on hospital readmission were quite different from that on length of stay. In general, readmission is partially affected too early discharge (28), when the final diagnosis and/or treatment were not reached. The COVID-19 pandemic decreased the hospital's general caseload due to the lockdown process and people who were generally scared of contracting the infection. Reduction of the general caseload could reduce the need for early discharge of the patients to reduce hospital congestion reduction of early discharge, thus reducing the chance of readmission.  Paragraph 9  **Before revision**  Prolonged LOS consistently increased during and post-COVID-19 pandemic peak for COPD because COPD shares similarities with COVID-19 (36,37), indicating potential challenges in managing these conditions. Cancer demonstrated a consistent decrease in prolonged LOS, suggesting improvements in the efficiency of care for this disease. The findings for CVD and DM exhibited mixed directions, highlighting the complexity of the pandemic's influence on hospital care across various health issues. This result related to the previous study in the USA that cardiovascular complications are a common manifestation of acute and post-acute COVID-19 infection (38).  **After revision**  Prolonged LOS consistently increased during and post-COVID-19 pandemic peak for COPD, potentially due to the similarities in clinical presentation between COPD exacerbations and COVID-19 (36,37). This overlap in symptoms may have led to challenges in differentiating between the two conditions, potentially delaying appropriate treatment and contributing to longer hospital stays. Additionally, the pandemic may have strained healthcare resources, limiting the availability of specialized care for COPD patients and further contributing to prolonged LOS (38).   e. We added the new paragraph in the last part of the discussion section for explained the results' implications   **After revision**  The results of this study have several implications for healthcare policy and resource allocation in Thailand: prioritizing cases for vulnerable populations, enhancing healthcare infrastructure, optimizing resource allocation, and strengthening pandemic preparedness. |
